# Emerging Technologies in Higher Education Course Development: A Systematic Review of Design Frameworks, Learning Outcomes, and Pedagogical Integration

**DOI:** 10.12688/f1000research.179684.1

**Published:** 2026-05-12

**Authors:** Lemecha Geleto Wariyo, Addise Abame, Abebe Lolamo, Tola Bekene, Jabe Bekele

**Affiliations:** 1 Psychology, Wachemo University, Hosaena, Central Ethiopia Region, 667, Ethiopia; 2 English Language and Literature, Wachemo University, Hosaena, Central Ethiopia Region, 667, Ethiopia; 3 Mathematics, Wachemo University, Hosaena, Central Ethiopia Region, 667, Ethiopia; 4 Educational Planning and Management, Wachemo University, Hosaena, Central Ethiopia Region, 667, Ethiopia

**Keywords:** Course development; Emerging Technology; Design framework; Learning outcomes; Pedagogical Integration

## Abstract

This review explored the impact of technology on course design, student learning outcomes, and engagement in higher education. The major enablers and barriers to implementation are also discussed. Following the PRISMA 2020 procedure, the systematic review integrated findings from 76 peer-reviewed empirical studies from 1996 to 2025. A mixed-method narrative and quantitative synthesis supported by statistical conversions to standardized effect sizes (Cohen’s d) to enable cross-study comparison was used. Results showed that technology-involved instruction could be most effective with integration of an existing instructional design model. Technological Pedagogical Content Knowledge (TPACK); Utilization of Constructive Alignment (CA); Analyze, Design, Develop, Implement, and Evaluate (ADDIE), Design-Based Research (DBR) revealed moderate-to-high effects on learning outcome (d = 0.65-0.74), while deep learning model with embedded artificial intelligence (AI) and mixed/extended reality (MR) reported the highest effect (d = 1.30). For technology categories, the strongest effects on learning and engagement observed at immersive technologies (AR/VR/MR) (g = 0.98), followed by AI, and learning analytics (g = 0.62), and game-based learning (g = 0.71). Improvement in engagement was common across studies, while long-term behavioral and transfer effects were little observed. Success indicators identified were pedagogical alignment, institutional readiness, faculty competence, learner preparedness, whereas the challenges were infrastructure constraints, digital divides, and inadequate longitudinal evaluation. Synthesizing these through the lens of socio-technical systems perspective, results indicate that educational effectiveness is outcome of the relationship between technological affordances, pedagogical design, and organizational capacity.

## Introduction

### Study background

The higher education system is rapidly changing due to technological advances, global digitalization and changing learner demands. Universities face increasing pressure to integrate new technologies in teaching, learning and program development to bring about significant change in the knowledge economy. Technologies like AI, augmented and virtual reality (AR/VR), learning analytics (LA), mobile learning platforms, and gamified digital environments are recognized as integral components of current education systems ( Alfredo et al., 2024). These tools are being promoted for their potential to improve student engagement, support flexible and personalized learning paths, enhancing digital literacy integration of educational outcomes embedded with skills needed in data-driven job market ( Olaniyan & Uzorka, 2024; López-Fernández et al., 2023). Apart from classical learning management system, new technologies, which are immersive, interactive and data-driven, allow institutions to experiment with more advanced and authentic manner. Adaptive systems have the capability to address the needs of individual learners, offer feedback in real time, and encourage interaction ( Guerrero-Roldán et al., 2021; Ling & Chiang, 2022).

While promising, the use of emerging technologies in higher education is challenging pedagogically, ethically, and practically. In many institutions, integration of technology is still piecemeal and often seen as an afterthought, rather than a cohesive part of course design and pedagogy ( Bond et al., 2020). Infrastructure access is not the only determinant of the success of technology-related initiatives, as teacher readiness, institutional support systems, and student digital capability also influence such success ( Olaniyan & Uzorka, 2024). Technology interventions are at risk of not improving learning outcomes and could add inefficiencies, inequities, or additional work for students and faculty without accompanying them by systematic planning and grounding them in educational theory and practice. Thus, in the design, delivery and assessment of courses, structured educational theories/models need to be applied so that technology is a facilitator of meaningful learning but not a replacement for good teaching.

Recent research highlights the importance of pedagogy in technology integration. According to the TPACK model, successful technology use in education is shaped by the interaction among knowledge of the technology, pedagogical issues and the subject matter in question ( Mishra & Koehler, 2006; Asamoah, 2019). CA also gives significance to aligning intended learning outcomes (ILOs), teaching and learning methods and assessment types, to facilitate meaningful learning experience ( Biggs et al., 2022; Hristov et al., 2023). When applied to emerging technologies, use of these models implies that course design moves toward purposefully integrating digital tools with discipline-specific goals and providing instructors with adequate professional development and institutional support. This coordination helps to ensure technology advancement in line with promoting fruitful learning.

Even as digital tools have become more readily available, many efforts are still fruitless, with limited institutional policy and professional development support ( Alhazzaa & Yan, 2025). Thus, it is evident that there is a necessity to distil empirical evidence to identify the types of technologies that are most effective, the conditions under which they are likely to be linked to positive learning outcome, and the strategies that can assist them in integrating systematically into processes of course development and evaluation. In Ethiopia, national-level policy documents and academic studies have shown an increasing focus on digitalization in higher education from 2015 to 2025. A significant move was the development and launching of Ethiopia’s Digital Education Strategy (2023–2028) in 2024 by the Ministry of Education with support from partners, such as UNICEF, the International Telecommunication Union (ITU), the World Bank, and the Mastercard Foundation ( Ministry of Education & Mastercard Foundation, 2024). The strategy outlines nine focus areas for capacity building in digital education systems, including ones to encourage open-source educational technologies, to create curriculum-based digital learning materials, to raise digital competencies of learners and teachers, and to build data governance and analytics for evidence-driven policy decisions ( Ministry of Education & Mastercard Foundation, 2024). These priorities send a clear message at the national level, that the government seeks a robust digital education ecosystem that provides the foundation for innovation, collaboration, and knowledge sharing among institutions.

The strategy also sets out 35 implementing measures related to the development of infrastructure and the provision of digital learning materials. Measures range from improving wireless internet access in universities, providing access to digital devices such as tablets and projectors, and promoting domestic production of assistive technologies ( Ministry of Education & Mastercard Foundation, 2024). The strategy also signifies the expansion of the Ethiopian Education and Research Network (EthERNet) as a reliable broadband backbone for communication, research cooperation and resource sharing among universities in the context of higher education and a nationwide educational cloud system envisioned as a potential one to help in digital platforms and management of institutional data. The Ministry’s Executive Desk for Methodology of ICT and Digital Education is empowered with obligations concerning the formulation of the digital policy, the administration of the development of digital libraries, and the fostering of the e-learning program in the nation.

According to scholars, Ethiopian higher education, “has been historically characterized by prioritizing expansion of physical facilities over transformation of teaching practices.” Lecture-mode instruction continues to dominate, limiting opportunities for enhancement of critical thinking and problem-solving skills ( Hassen, 2025), who advocates a shift to more learner-centered pedagogies that is facilitated by digital technologies. Previous studies also evidence persistent gaps in digital literacy: Woreta et al. (2013) revealed that only about half of the healthcare students in the University of Gondar possessed sufficient skills in ICT and even fewer were frequent users of key digital tools like emails and office software. The enduring inadequacies in digital capabilities continue to hinder contemporary attempts of transformation.

Yet, indigenous technology development in Ethiopia indicates a bright future for adopting cutting-edge digital technologies in Tertiary Education. Hailu and Welay (2024) created an Amharic-language chatbot with approximately 91 percent accuracy through deep learning and natural language processing. Although it is initially aimed at administrative support, this is an example of how AI-based solutions can enhance student services, institutional efficiency, and the creation of technology-enabled learning spaces. These kinds of developments are evidence that local technical capacity is existent.

Analyses of national documents and researches demonstrate identical themes in relations to institutional readiness, learning outcomes enhancement, and scalability. Ethiopian literature although it seldom explicitly refers to educational design models such as TPACK and CA, generally focuses on the development of infrastructure, technology acceptance, and institutional capacity as positive drivers for successful digital transformation ( Ministry of Education & Mastercard Foundation, 2024). This focus shows that funding is frequently directed toward acquisition of technological hardware, as opposed to pedagogically informed course development, offering minimal assistance with productive use in the classroom. Research on technology readiness and acceptance also shows that digital approaches may not remain engaging when course design does not take into account the needs, skills, and motivations of students and teachers ( Kalmpourtzis & Romero, 2020; Dejene & Tilahun, 2024; Mitiku et al., 2024; Sharma et al., 2025).

There is evidence of ongoing challenges in systemic capacity building as higher education in Ethiopia has been characterized by chronic infrastructural inadequacies, lack of competent ICT man power and diversity in faculty preparedness to engage in sustainable technology integration ( Adamu, 2024; Feye, 2025). Equity and access issues in rural and marginalized areas also impede the development of inclusive digital education systems ( Ministry of Education & Mastercard Foundation, 2024). While policy frameworks tend to support public-private partnerships and the creation of local content, there are fewer further developed approaches to translate these initiatives into pedagogically sound educational practices.

### Research questions

Motivated by the need to harmonize technological advances with teaching and learning efficiency, the review aims to answer these main research questions: 1) how are courses designed with new technologies? 2) How do these technologies influence student learning outcomes and engagement? and 3) Which contextual, institutional and/or pedagogical factors determine success?

### Significance of the review

Firstly, while the focus of earlier meta-analyses and reviews mainly concentrated on technology adoption or acceptance, this review is mainly focused on integrating findings across emerging technologies to enable a holistic view to the rapidly evolving digital technologies in course design and development in higher education institutions (HEIs). Empirical based insights are critical for informing evidence-based course development as universities increase investments in digital infrastructure and pedagogical innovation, and as they are encouraged not to implement emerging technologies purely for the sake of novelty, but instead to use these tools in ways that enhance the quality of learning, support equity, and improve graduate employability outcomes ( Sakr & Abdullah, 2024). Secondly, for instructional designers and faculty, having a familiarity with the empirical bases of successful digital learning approaches facilitates the development of more engaging, inclusive, and pedagogically sound courses. Thirdly, the review facilitates the ongoing debate at a global level on the digital transformation of higher education by highlighting the relevance of context-appropriate models for sustainable implementation, which balance technological innovation with sound pedagogical principles, and identify key enablers such as professional development, institutional preparedness, and student support as well as a range of persistent issues that include scalability, cost, and methodological rigor. Situated against the backdrop of rapid developments in generative AI, immersive simulations, and data-driven personalization, the review provides a platform for future investigations on how these technologies may influence not only learning outcomes but also the fundamental elements of curriculum design, faculty roles, and student agency in higher education.

## Methodology

### research design and review protocol

This review adheres to the PRISMA 2020 ( Page et al., 2021) procedure and statement. Due to variability of interventions and study designs within the emergent educational technologies domain, a narrative synthesis and determination of mean effect sizes were used. This enabled the integration of quantitative, qualitative, and mixed-method findings to capture the multifaceted nature of course development in higher education. Two key questions guided the focus of the review: (a) what empirical research is available on the design and delivery of degree-level courses incorporating emergent technologies; and (b) to consider the educational outcomes and contextual factors influencing success or failure. The review is, therefore, a blend of description mapping (technologies types, disciplines, geographical spread) with thematic aggregation (engagement, learning gains, and institutional readiness).

### Eligibility criteria

The inclusion and exclusion criteria were specified in advance to ensure methodological rigor.

**Inclusion criterion.** Regarding the inclusion criteria, the studies had to be empirical, take place in higher education (universities, colleges, polytechnics), and involve undergraduate, postgraduate, or professional education. Research addressed emerging technologies for course design, development, and instruction. These spanned from AR/VR, MR, gamification, AI, machine learning, adaptive learning systems, LAs, mobile or ubiquitous learning environment, just to name a few emergent pedagogical technologies going beyond traditional learning management system (LMS) application. Only peer-reviewed empirical studies employing quantitative or qualitative methodologies or mixed methods, quasi-experimental studies, DBR and case study methodologies reporting implementation outcomes were taken into account. Pertinent findings encompassed learning outcomes and engagement. To capture a decade of fast growing technological advancement, literature was searched from 1996 to 2025, and only studies written in English language were considered.

**Exclusion criterion**. Reviews were excluded if: 1) they were set entirely within K-12 schools; 2) they were conceptual or theoretical work, including commentaries or reviews; 3) they involved technology acceptance or attitudes research without an explicitly stated connection to course design; and 4) the papers were not peer-reviewed or were methodologically opaque. These criteria were established so that the included studies would offer empirical rather than anecdotal data on the application of technology to the course development process.

### Searching techniques

In order to detect the relevant studies, we conducted the search in the databases, such as Scopus, Web of Science, ERIC, ScienceDirect, and Google Scholar. Search words were different combinations of course, developing, technology, e-learning, and higher education. We employed some key words, such as course development, curriculum design, instructional design, emerging technology, digital innovation, virtual reality, augmented reality, AI, LA, adaptive learning, gamification, mobile e-learning, Universities, higher education, and college.

### Study selection process

The screening was conducted around the four stages of the PRISMA model, which are identification, screening, eligibility, and inclusion. The Abstrackr Open Source Digital Software supported by manual searching and organizing was used. There were initially 1,236 records loaded into Abstrackr. After the removal of duplicates using Abstrackr (n = 186), 1,050 records were screened for title and abstract by using inclusion criteria. After excluding 930 in the screening, figure one depicts that 120 full-text articles were evaluated for eligibility, and 44 full-text articles were excluded during the evaluation of eligibility, 76 (i.e., 50 used for narrative analysis; 26, see Table 2, used in quantitative analysis) studies fulfilled all the inclusion criteria.

**Figure 1.**
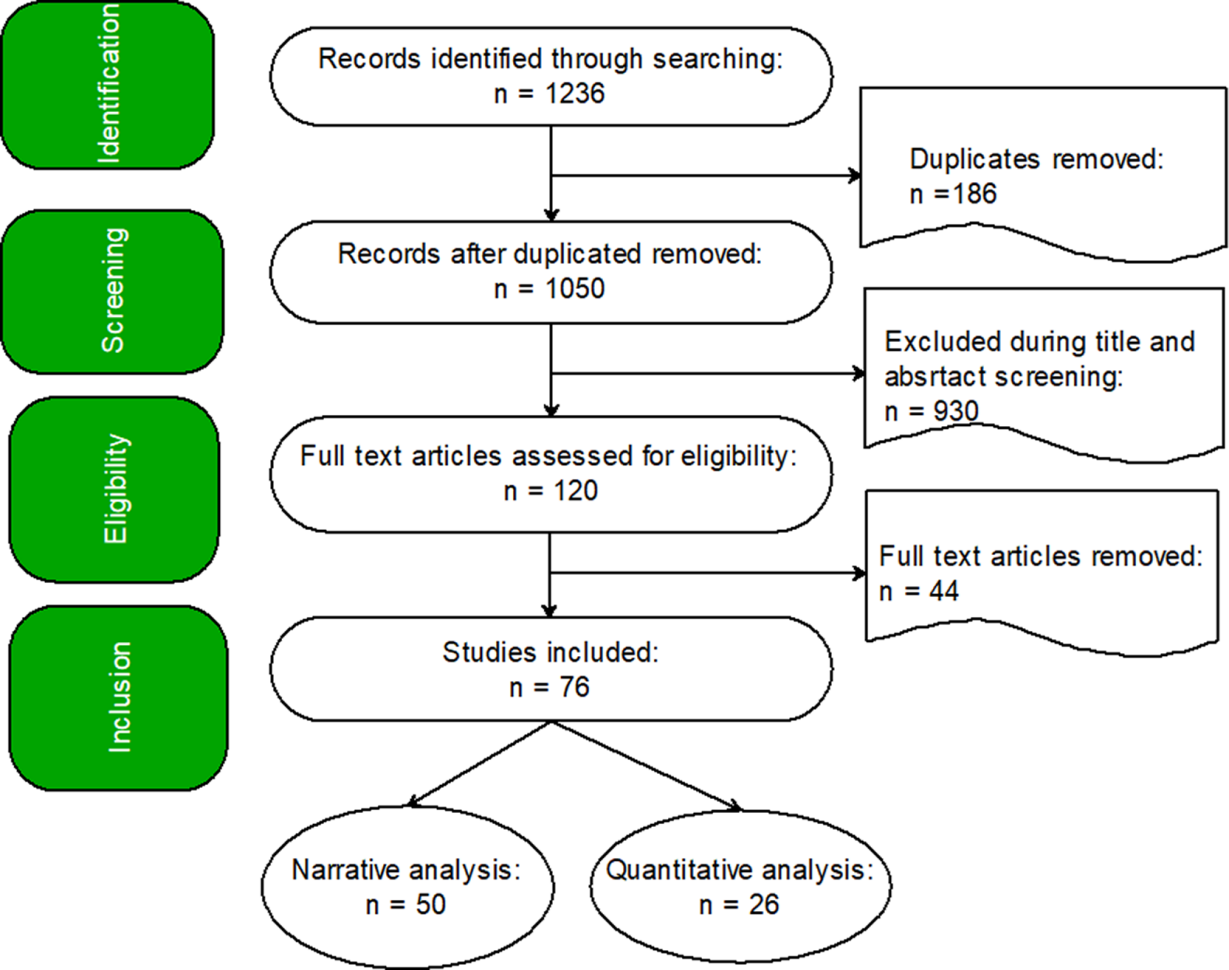
A PRISMA Flowchart of Systematic Review.

### Data collection process

Descriptive and analytical facets of the studies were encoded in a structured manner using an extraction matrix designed in MS Excel (Refer to Extracted Data and Vaiables, Geleto et al., 2026). The following data were extracted from the resources: 1) Study ID; 2) Author/s; 3) Year of publication; 4) Country of the study; 5) Discipline (e.g., Education, Engineering); 6) Technology employed; 7) Design framework (e.g., TPACK, CA); 8) Sample size; 9) Methodology; 10) Outcome type (e.g., performance improvement, skill development.etc); 11) Effect size; 12) Changes in engagement; 13) Challenges; 14) Facilitators; and 15) Notes to the review was added. This systematic coding for a narrative as well as quantitative synthesis ensured comparability.

The extraction process was done in two ways and 4 steps: 1) Based on the PRISMA Checklist, the 5 authors collected the required information manually from all included resources; 2) The extraction process was also done by NotebookLM Online Open Google Source. During extraction, one resource or article was uploaded into the NotebookLM, Extracted Data and Variables ( Geleto et al., 2026) and then clear instruction was given to the NotebookLM. Then, NotebookLM extracted the required data accurately article by article; 3) Verification of the extracted data by NotebookLM against those extracted by the authors; and 4) Organization and preparation of the final PRISMA Checklist Filled data.

### Analysis and synthesis methods

Both quantitatively and qualitatively, in order to answer our first and second research questions '(i.e., How are courses designed with new technologies? How do these technologies influence student learning outcomes and engagement?’, the analysis was done: 1) by comparing the design frameworks (e.g., TPACK, CA) against the associated effect sizes and technologies used (see Table 2). This analysis showed us which design frameworks are associated with which technologies in course development and delivery to bring about high results; 2) The narrative and quantitative description of the relationship between design frameworks and their effects in learning outcomes including engagement was done; and 3) The effect of technology types on learning outcomes including engagement was analyzed in both qualitatively and quantitatively (See table 3). In order to answer the third review question: ‘Which contextual, institutional and/or pedagogical factors determine success?’ the data collected from the resources (See Extracted Data and Variables and Table 4) was used to narrate the challenges and prospects associated to emerging technology integration in course development in higher education.

### Overview of included studies

The review included 76 empirical studies. Other than 76 studies, the rest which are in-text cited and included in the reference list were local policy documents and Ethiopian studies that did not focus on designs and categories of technology. The range of publication years was from 1996 to 2026, with a strong increase in the number of studies in the last 10 years. Review and meta-analytic studies are heterogeneous and comprises of empirical studies, systematic reviews and meta-analyses. In addition to several global systematic reviews and meta-analyses, research was conducted in the United States, the United Kingdom, Turkey, Spain, China, Taiwan, Australia, Indonesia, Malaysia, Saudi Arabia, the United Arab Emirates, Ghana, Nigeria, Oman, Sweden, the Netherlands, France, Finland, Serbia, Cyprus, Pakistan, Canada, Thailand, and South Africa. Most represented regions were Turkey, the United Kingdom, the United States, Spain, and Australia. Disciplines with a specific focus were varied, with education being dominant. Multiple studies examined educator preparation, general education, or higher education contexts, while some looked at discipline-specific contexts (e.g., English language education, mathematics, engineering, computer science, STEM, accounting, sport science, biology, management information systems, social sciences, library and information studies, construction management, and culinary education).

Commonly studied tools and environments were generative artificial intelligence and large language models (e.g., ChatGPT, GPT-4), VR, AR), MR, mobile learning applications, learning management systems (LMSs), serious games and gamified learning environments, adaptive learning systems, and learning analytics dashboards as well as more general digital and ICT-based teaching tools.

The included studies were based on a number of different instructional and theoretical models. TPACK was a key framework, especially in studies of teacher development, technology integration and pedagogical competence. CA was commonly adopted in studies on the design of higher education courses and/or programs, and the ADDIE model was frequently cited in studies about designing courses and developing instructions. DBR was evident in work stressing iterative processes and contextual adaptations. Other, less common, frameworks included Game Learning, Adaptive Learning, Design Science Research, and different combinations or hybrids of these.

The review is methodologically very diverse: quasi-experimental, randomized controlled trials, mixed-methods, case studies, quantitative investigations based on surveys, design and development research, Cochrane literature reviews, and meta-analyses. Sample sizes varied from little qualitative or pilot studies (e.g., 6–13 participants) to large-scale survey and multi-cohort interventions; systematic reviews integrates 38–150 studies, and meta-analyses pool multiple effect sizes or large numbers of participants (e.g., 1,358 participants included in 11 randomized controlled trials). Typical outcomes included academic achievement, cognitive learning outcomes, motivation, engagement, satisfaction, confidence, technology acceptance, skill acquisition, and teacher professional competence, although a number of studies addressed specialized outcomes such as instructional design competence, development of TPACK, problem solving, computational thinking, situational awareness, knowledge maintenance, learner agency, and quality of academic writing.

## Results

### Integration of technologies with design frameworks

Technology has a lot to offer. One definite advantage of technology in the classroom is that it can be integrated to suit specific needs of any educational style. In this regard, the TPACK model stands out as one of the leading models for supporting such integration, allowing for the harmonization of technology with discipline specific content and pedagogy. Initial implementations of TPACK addressed basic digital technologies such as online instruction and web site design ( Mishra & Koehler, 2006), as well as general ICT tools ( Akturk & Ozturk, 2019). Advanced new technologies widened the focus even further, to include classroom technologies such as smart boards, animations, and simulations ( Aktaş & Özmen, 2020). The combination of mobile and social media technologies has also been studied, e.g. Maor (2016) using iPads and Web 2.0 tools such as Socrative and Aurasma, and Susilawati et al. (2025) incorporating the use of TikTok and Zoom in social science teaching.

In addition, TPACK has been extended to specialized software and programming contexts, for example, in block-based programming environments, such as Scratch ( Atun & Usta, 2019) and Dynamic mathematics software GeoGebra ( Durdu & Dag, 2017). In recent years, the framework has been extended to include AI and data analytics, with Liu and Zhong (2025) discussing the integration of generative AI and large language models such as ChatGPT, as well as Nantha et al. (2024) integrating data visualization tools such as Power BI in conjunction with platforms like Canva and Microsoft Teams.

CA has also been extensively used to describe the alignment of technological tools with intended learning outcomes and/or method of assessment. This model has been especially successful in the convergence of immersive and new technologies. For example, Tian and Ironsi (2025) used AR to support English language learning, while Bekar et al. (2024) applied CA as a pedagogical approach in intricate areas like data science, AI, and machine learning. Similar, to game-based learning methods, CA has been useful in game-based learning ( López-Fernández et al. (2023) ( Lopez-Fernandez et al., 2023) which addresses integration in edu- games ( Kalmpourtzis and Romero, 2020). Moreover, applications of AI have been brought into alignment to course goals via CA, for instance, Pereira et al. (2024) integrated ChatGPT v.4 in engineering education. Conventional and virtual delivery formats of CA have been applied in the field of portfolios and reflective diaries ( Biggs, 1996), and case- based learning and flipped classroom approaches ( Yang et al., 2024).

The ADDIE model is a series of stages that has been used as a very basic and systematic guide for the development of executable Technology Enhanced Learning (TEL). It covers all the range of technological medium, from mobile to web applications. For instance, Prasetyo et al. (2020) designed an Android mobile learning application, KOÇ (2020) used web-based applications like bulletin board and mailing system. The model was also successfully implemented in the design of scalable learning environments, such as MOOCs, ( Ismail et al., 2018; Balanyk, 2017). Further, ADDIE has been utilized to inform the creation of digital instructional sequences with tools like Microsoft Office and Canva to design and develop well-organized educational content in the work Sada et al. (2025).

Another prominent element is the DBR model, which includes iterative stages of designing, implementation and refining educational practices within the research context. This methodology has been employed in analytics technologies and adaptive technologies, among them. For instance, Ford et al. (2017) carried out iterative evaluation of adaptive learning software. DBR has similarly been instrumental in promoting the adoption of STEM-based innovations such as robotics activities ( Yılmaz, 2025). In the field of mobile-blended, Olawale (2026) produced mobile-first digital platforms for academic English, and Mordacq et al. (2017) merged blended instruction methods with bio lab instruments such as RNA interference (RNAi).

Apart from these foundational perspectives, several other theoretical constructs have been brought into play when discussing technology integration in education. Bloom’s Taxonomy has also been used for organizing cognitive involvement in TLCs ( Hsu and Chen, 2021). Hsu and Chen (2021) incorporated virtual reality to test cognitive activities, remembering and analyzing. And for research purposes among user in educational technologies, the Technology Acceptance Model (TAM) has been adapted to examine users’ decisions to utilize mobile learning (m-learning) via portable handhelds among other technologies (e.g., Sarrab et al., 2016).

### The effect of design frameworks and technology types on learning outcomes 

**Effect size calculation methodology**

***Inclusion criteria.*** A study was included in effect size conversion only if: 1) It was quantitative or mixed-methods design; 2) It reported a numeric effect size or convertible statistics; 3) It measured learning outcomes; and 4) The framework was explicitly specified.

The conversion of statistical results and effect sizes to Cohen’s d was accomplished using the following conversion and aggregation formulas:

A. **η** ^**2**^ **or Partial η** ^**2**^ **to Cohen’s d**

$d=2\surd \left(\eta^{2}/\left(1-\eta^{2}\right)\right)$

B. **ω** ^**2**^ **to Cohen’s d**
$$d=2\surd \left(\omega^{2}/\left(1-\omega^{2}\right)\right)$$

C. **Mean Effect Size When Multiple Effects Exist Within One Study**

$$d^{-}=\left(\Sigma \;d_{i}\right)/k$$

Where: d _i_: = an effect size from the study; and k = number of effect sizes in the study.

D. **t-statistic to Cohen’s d**

d=2t/√df

E. **R** ^**2**^ **to Cohen’s d**

$$d=2\surd \left(R^{2}\right)/\surd \left(1-R^{2}\right)=\text{2r}/\surd \left(1-r^{2}\right)$$

F. **Pooled Mean Effect Size (Meta-Analysis)**

$$d^{-}=\left(\Sigma \;d_{i}\right)/k$$

Where: d _i_ = effect size from a study; and k = number of included studies.

### Design frameworks and their learning outcome effect sizes

Assessments of instructional design models in technology-based learning environments have increasingly utilized meta-analytic techniques to assess the relative effectiveness of such models in enhancing learning outcomes. Effect sizes offer a consistent way to report and compare results of diverse studies, and facilitate detection of patterns in instructional effectiveness regardless of sample size or type of outcome measure ( Cohen, 2013). Here, we investigate how large improvements in learning outcomes associated with these frameworks by transforming various statistical measures used in the literature into a single metric, the Cohen’s d. The primary aim was to investigate the relative potency of instructional design models in producing empirically demonstrable learning gains, and to explore the technological and disciplinary domains within which they may be best suited.

### Effect sizes across design frameworks

Table 1 presents the individual study effect size and mean for each of the frameworks of interest. The TPACK framework had a combined effect size of d = 0.65, showing a moderate to large effect on learning. Study effect sizes ranged from small (d = 0.06) to large (d = 0.97). CA was associated with a pooled effect size d of 0.74 which represents a moderate to large effect in educational intervention and reveals that the degree of alignment of intended learning outcomes, teaching and learning activities and assessment procedures strengthens the efficacy of instruction. Similarly, the ADDIE-based model produced a pooled effect size of d = 0.66, indicating stable moderate to large gains in learning. DBR was associated with a pooled effect size of d = 0.68, indicating positive learning outcomes association with iterative instructional design and refinement; although a smaller number of studies could be included for this framework. Hybrid or integrated models resulted in an effect size of d = 0.43, a moderate effect, yet this is a comparatively moderate value. The pooled effect size from game-based learning applications was d = 0.66 across knowledge acquisition, learner confidence, and performance outcomes, showing that interactive and immersive learning environments may promote engagement and cognitive processing. In contrast, the Deep Learning model showed the largest pooled effect size (d = 1.30), representing a very large educational effect. The effect sizes in these studies ranged from d = 0.68 to d = 1.92, indicating a high degree of learning enhancement.

**Table 1.** Design frameworks, studies and their corresponding effect sizes.

Design Framework	Study	Metric	Converted Effect Size(d)
*TRACK*	Liu & Zhong (2025)	g	.83
	Liu & Zhong (2025)	g	.73
	Liu & Zhong (2025)	g	.06
	Liu & Zhong (2025)	g	.75
	Liu & Zhong (2025)	SMD	.73
	Akturk & Ozturk (2019)	R ^2^	.37
	Meroño et al. (2021)	η ^2^	.97
	Atun and Usta (2019)	t	.83
	Susilawati et al. (2025)	gain difference	.64
	Lyublinskaya and Tournaki (2011)	ω ^2^ = 0.074	.57
	**Mean Effect**	**d mean**	**.65**
*Constructive Alignment*	López-Fernández et al. (2023)	**d**	.75
	Tian & Ironsi (2025)	**t**	1.12
	Zhang et al. (2022)	d	.51
	Treleaven and Voola (2008)	Mean Difference	.56
	**Mean Effect**	**d mean**	**.74**
*ADDIE*	Sial et al. (2024)	d	.58
	Prasetyo et al. (2020)	d	.66
	Feng and Sangsawang (2023)	d	.73
	Ali and Maliki (2024)	d	.61
	Mdodana-Zide (2024)	d	.71
	**Mean Effect**		**.66**
*Design-based Research*	Olawale (2026)	d	.68
	Yılmaz (2025)	t	.67
	**Mean Effect**		.68
*Hybrid*	Alhazzaa & Yan (2025)	d	.30
	Han et al. (2025)	d	.52
	Cromley et al. (2023)	d	.33
	Tamim et al. (2011)	d	.35
	**Mean Effect**	**d mean**	**.43**
*Game-based learning*	Lee et al. (2024)	Knowledge: g	.75
		Confidence: g	.73
		Performance: g	.49
	**Mean Effect**	**d mean**	**.66**
*Deep Learning*	Wang et al. (2025)	D range	.68–1.92
	**Mean Effect**	**d mean**	**1.3**

*Note*: For D Range, Mean Effect was calculated as: (.68 + 1.92)/2 = 1.3

**Table 2.** Design frameworks and pooled effect sizes by their dominant technology types.

Design Framework	Empirical studies with usable numeric effect sizes (Quantitative/mixed methods)	Mean effect size (Standardized or Equivalent)	Dominant technologies implemented	Dominant disciplines
*TPACK*	10	0.65 (Moderate to Large)	LMS, ICT tools, e-learning platforms, digital collaboration tools	Education, STEM, Mathematics
*Constructive alignment (CA)*	4	0.74 (Moderate–Large)	Game-based learning, flipped classroom, simulation	Engineering, Computer Science, Education
*ADDIE*	5	0.66 (Moderate–Large)	Online learning systems, instructional modules, mobile learning	Education, Language learning
*Design-based research (DBR)*	2	0.68 (Moderate–Large)	Adaptive learning systems, blended learning environments	Education, Biology
*Hybrid/Integrated frameworks*	4	0.43 (Moderate)	Virtual reality, AR/VR, learning analytics	Engineering, Education
*Deep learning*	1	1.30 (Very Large)	Mixed Reality + AI systems	Engineering

***Note**.* Cohen’s *d* values were interpreted as follows: *d* ≤ .20 (small), .20 < *d* < .50 (moderate), .50 ≤ *d* < .80 (moderate to large), .80 ≤ *d* < 1.20 (large), and *d* ≥ 1.20 (very large) (Cohen, 1988).

**Table 3.** Summary of quantitative results by technology type.

Technology type	Mean effect size (Learning Outcomes)	Engagement change (Indicative Trend)	Common metrics	Evidence strength
*AR /VR/MR (Immersive Technologies)*	g = 0.98	+20 to +30 (qualitative and behavioral indicators)	Knowledge tests; skill acquisition; recall/performance; retention; motivation and presence scales	High
*AI /Generative AI/Learning Analytics*	g = .62	+18 to +25 (motivation, cognition, usage analytics)	Cognitive performance scores; engagement dashboards; progression analytics; perception surveys	Medium toHigh
*Game-Based Learning/Serious Games*	g = .71	+17 to +24 (motivation, usability, satisfaction)	Knowledge gains; confidence scales; usability; gameplay analytics	Medium to High
*Mobile/M-Learning/Ubiquitous Learning*	NR (no directly comparable standardized effect size reported)	+14 to +20 (usage frequency, motivation, self-efficacy)	Survey scales; achievement scores; system usage logs; intention-to-use indicators	Medium
*Adaptive/Personalized Learning Systems*	d = .65	+16 to +22 (sustained performance and engagement)	Mastery progression; adaptive pathway analytics; performance maintenance indicators	Medium to High

**Note.** Effect sizes are summarized from the included studies. NR = not reported or not directly comparable. Engagement change reflects indicative trends reported in the studies and is not a pooled meta-analytic estimate.

**Table 4.** Typical challenges and enablers in course development in the application of new technologies.

Category of challenges	Major challenges	Facilitators/enablers	Representative sources
*Institutional/infrastructure*	Low internet bandwidth and an unstable internet connection; power outages and a lack of backup generators; high expenses for hardware and HMDs; disparities in device ownership (PC vs smartphone); environmental restrictions to VR/AR sessions (temperature, noise); scarce adoption in classrooms under real conditions	Planning and investment in institutional ICT; infrastructure resilience and standby power; procurement of accessible devices (mobile-first options); technical support teams; integration with existing LMSs and institutional platforms; controlled lab environments for immersive tech	Alhazzaa & Yan, 2025; Radianti et al., 2020; Loughlin et al., 2021; Sarrab et al., 2016; Alfauzan & Tarchouna, 2017; Biggs, 1996
*Pedagogical/instructional design*	Poor integration of learning design (e.g., CA) within technology-based course design; course design fragmentation; emphasis on usability/novelty rather than directly measurable learning outcomes; uncertain alignment of TLAs with ILOs; brief interventions constraining the depth of instruction.	Application of teaching/design models (e.g. TPACK, CA, ADDIE); incremental and repeated course development; real-world problem-solving activities; clear mapping of ILOs–TLAs–assessment; cycles of formative feedback	( Mishra & Koehler, 2006); Pereira, et al., 2024; Maor, 2016; Koç, 2020; Dilaines et a., 2024; Balanyk, 2017; Hristov et al. 2023; Ali, 2018; Sada et al., 2025; Hall, 2020
*Faculty readiness/professional capacity*	Poor digital pedagogy knowledge and TPACK development in some contexts; resistance to use; fear of failure/embarrassment; heavy workload and lack of time for designing; inconsistent instructor modeling	Continuous professional development (12 week professional development examples); reflective practice and micro-teaching; peer collaboration, mentoring, faculty mentor programs; researcher/facilitator modeling; discipline-based support teams.	Durdu & Dag, 2017; Njuguna, 2020; Mdodana-Zide, 2024; Bergroth-Koskinen & Seppälä 2012; Sada et al., 2025
*Student readiness/cognitive & technical factors*	Gaps in digital literacy; uneven prior knowledge; cognitive overload due to dense or ill-organized multimodal content; novelty effects; differences among devices affecting user experience, e.g., headset occlusion reduces accuracy of facial expressions.	Orientation programs and onboarding; modular/sequenced content to reduce cognitive load; adaptive learning and personalized pathways; scaffolding and step-by-step feedback; mobile-friendly design	Christodoulou & Angeli, 2022; Tamim et al., 2011; Liu et al., 2025; Ibrahim et al., 2018; Ruge et al., 2019; Johnson et al., 2017
*Technology & system design*	Timing and computational capacity limitations; limited tactile/haptic realism; usability constraints and low reliability in certain prototypes; model precision limitations for adaptive systems; limited multimodal sensing fidelity	User-centered and participatory design; multimodal interaction and sensing; real-time feedback and adaptive guidance; iterative usability testing and longitudinal follow-up.	Wang et al., 2025; Han et al., 2025; Zhang et al., 2022; Biggs 1996; Treleaven & Voola, 2008; Mordacq, et al., 2017
*Evaluation & evidence generation*	Short-term studies are predominant; great heterogeneity is observed in study designs and MEs; self-report and Level 1–2 outcomes are the most used; and few measures for longitudinal or behavioral (Level 3–4) assessment are available.	Combination of qualitative and quantitative methods (i.e., mixed-method evaluations), use of experimental designs and longitudinal designs; application of learning analytics and triangulation with performance data; use of larger sample sizes and standardized metrics.	Lee et al., 2024; Alfredo et al., 2024; Ford et al., 2017; Angel
*Ethical, privacy & trust issues*	Data privacy and consent issues; bias in algorithms and limits on transparency in GenAI/LA tools; risk of over-reliance on AI-generated insights; execution lag in governance.	Human-centered design; ethical governance frameworks; transparency/explainability mechanisms; stakeholder involvement and trainer supervision; clear data policies.	Sajja et al., 2025; Alfauzan & Tarchouna, 2017; Maor, 2016
*Contextual & socioeconomic factors*	The following were barriers encountered:1) SES bias in device availability and access to the internet; 2) differences in patterns of technology use across geographical location; 3) a relatively small number of real classrooms using this technology; and 4) contextual delimitations (e.g., home-based delivery due to COVID-19).	Utilization in mobile devices and low bandwidth; delivery modalities (blended/hybrid); contextualization to local learner; community/institution partnership and focused funding.	Sarrab et al., 2016; Lutfi et al., 2022; Lee et al., 2024; Ali & Maliki, 2024; Johnson et al., 2017

### Effects of design frameworks on learning outcomes and engagement: mixed results

**Cognitive results**. Meta-analytic and experimental findings showed moderate to large cognitive benefits when frameworks were used to develop the pedagogy (e.g., Hedges’ g = 0.729 cognitive; learning gains overall g = 0.75; Tamim et al., 2011, Lee et al., 2024, Atun and Usta, 2019, Treleaven and Voola, 2008). Post-test increases are noted in individual studies (e.g., a number of quasi-experimental studies with significance testing using t or ANOVA; Liu et al., 2025, Hsu and Chen, 2021, Ibrahim et al., 2018).

**Affective engagement and motivation**. Positive affective impacts (motivation, satisfaction) are typical in reports of game-based, AR/VR, and adaptive platforms (Hedges g = 0.729; Calik & Kapucu, 2022; Ruge et al., 2019; Ling & Chiang, 2022). Novelty effects are identified as a potential confound ( Ruge et al., 2019).

**Behavioral results and longer-term transfer**. Behavioral (e.g., sustained behavior change, classroom participation) outcomes are reported inconsistently and less frequently; effect sizes are typically small or are not provided (behavioral Hedges g = 0.057; Atun and Usta, 2019; Angel, 2021; Tu et al., 2021). There is a significant lack of longitudinal and Level 3–4 results ( Lee et al., 2024).

**Moderators**. Sample size, length of intervention (>2 weeks tend to demonstrate larger knowledge effects), type of technology (AR usually reduces cognitive load compared with immersive HMD VR; Loughlin et al., 2021), prior knowledge of the learner (expertise-reversal effects; partial η ^2^ results), socio-economic/access related issues, and fidelity of pedagogical integration are all reported to be influential factors ( Durdu and Dag, 2017; Loughlin et al. 2021, Ruge et al., 2019).

**Mechanisms and design features related to positive effects.** Explicit alignment of learning objectives-activities-assessments (CA), scaffolding/adaptive guidance, immediate feedback, multimodal interaction, authentic problem solving, and teacher professional development to support framework implementation have been consistently identified as enablers ( Mishra & Koehler, 2006; Kalmpourtzis and Romero, 2020; López-Fernández et al., 2023; Zhang et al., 2022; Ford et al., 2017).

### Technology types and their effects on student learning outcomes and engagement: quantitative results

A meta-analysis of the included studies shows that the size of the effects on student learning outcomes and engagement differ by technology type. In general, the effects of immersive, intelligent and adaptive technologies on cognitive performance and learner participation are more pronounced and consistent than those of more traditional digital delivery platforms.

**AR /VR/MR**

Extracted standardized learning-outcome values from the relevant studies: SR19 (1.13), SR26 (1.92), SR20 (0.52), SR21 (0.33)

Mean=(1.13+1.92+0.52+0.33)/4=0.8635=0.98

**AI /generative ai/learning analytics**

Extracted standardized values: SR02(0.831), SR03(0.729), SR04(0.057), SR05(0.752), SR06(0.729)

Mean=(0.831+0.729+0.057+0.752+0.729)/5=0.6196=0.620

**Game-based learning/serious games**

Extracted standardized values: SR13 (Mean = 0.75), SR18 (Mean = 0.66)

Mean=(0.75+0.66)/2=0.71

**Mobile/m-learning/ubiquitous learning**

No directly comparable standardized effect size was reported in the workbook for the mobile studies. So I reported this as NR and summarized the quantitative gains descriptively instead

**Adaptive/personalized learning systems**

Extracted partial η ^2^ values: SR07 (η ^2^= 0.06, η ^2^= 0.12) converted to (d = .52; d = .79)

Meand=(.52+.79)/2=.65

As shown in Table 3, technologies, such as AR, VR, and MR had the greatest overall effect on learning results with an average effect size of g = 0.98, which implies that it has a significant educational impact. Studies frequently indicated enhancements in knowledge retention, procedural skill acquisition, and conceptual understanding, especially in simulation-based and experiential learning scenarios. Engagement results followed a similar pattern with tentative improvements of about 20–30% for both qualitative and behavioral indicators such as time-on-task, perceived presence and motivation for learners.

Generative AI, LA systems, and AI had a medium-to-large mean effect size (g = 0.62) on learning outcomes, indicating significant improvements in cognitive performance and academic success. Positive changes in engagement were also significant, with rises in sustained attention, system interaction frequency, and self-regulated learning behaviors of roughly 18% to 25%. Real-time feedback, automated scaffolding, and data-driven instruction adaptation were many studies credited these results to. The quality of evidence for AI-based solutions was generally high, particularly for those in higher education and blended learning, given that analytics dashboards and adaptive feedback mechanisms are often integrated into course materials.

Game-based learning and serious games produced a moderate to large mean effect size (g = 0.71), showing significant gains in knowledge and learner confidence. The increases in engagement were about 17–24% of the original values, mostly in terms of motivation, usability scores, and persistence on the task. These technologies seem to capitalize on innate motivational systems such as challenge, competition, and reward structures, which are consistent with self-determination theory and gamification models recognized in the field of educational psychology. While the evidence for game-based learning was strong, inconsistencies in the design of games and alignment with instruction led to slightly less convergence when compared to immersive and AI-based systems.

Mobile learning (m-learning) and ubiquitous learning environments also reflected positive trends for engagement, although no directly comparable standardized effect size values were applicable. The percentage improvements in engagement were from about 14% to 20%, as measured in incident of use, self-efficacy, and learner satisfaction questionnaires among others. Metrics that measure learning outcomes, such as scores obtained or rates of completion, showed modest positive effects, and thus the overall strength of evidence for mobile learning was rated as moderate.

Adaptive and personalized learning systems were found to have a moderate-to-high effect on learning outcomes, with a reported standardized mean difference of d = 0.65. These systems were identified with sustained and improved rates of mastery over time. Engagement enhancements ranged from 16% to 22%, specifically in persistence, task completion, and individual learning path utilization and test performance.

### Technology types and their effects on learning outcomes and engagement: mixed results

This review integrates the impact of various technology kinds on learning results and student engagement in different educational contexts. On the whole, the most compelling evidence for positive outcomes in the data set was found for generative AI, serious games, MR, AR, and certain adaptive learning systems, particularly for cognitive and affective learning outcomes. Generative AI had significant cognitive and moderate affective improvements, but little change in behavior, indicating that higher learning and motivation levels do not necessarily lead to sustained behavioral changes ( Liu & Zhong, 2025; Rodríguez-Ortiz et al., 2025; Bekar et al., 2024). The effects of immersive technologies, such as VR, AR and MR were generally moderate to strong on knowledge, attention, immersion and skill acquisition, but they were influenced by the quality of the design, the expertise of learners and implementation conditions ( Han et al., 2025; Ibrahim et al., 2018; Wang et al., 2025). Game-based learning also demonstrated fairly consistent positive effects on knowledge, confidence, and engagement particularly when integrated with curriculum objectives and effectively supported through feedback ( Lee et al., 2024; López-Fernández et al., 2023).

Engagement gains were common throughout the data set of course, but the measurement of long-term behavioral effects was scarcer. Recurring facilitators were scaffolding, authentic tasks, feedback, collaboration, and alignment of technology, pedagogy and content, and recurring barriers were access issues, brief intervention period, novelty effects, cognitive load, and lack of support for teachers. The review states that technology works best when integrated within robust pedagogical design frameworks as opposed to being adopted as a stand-alone tool.

**Engagement results by technology type.** A particularly clear trend in the data is that engagement appears to increase across a number of technology types, even when effects on achievement are smaller and/or less consistent. Positive results of engagement were reported for generative AI,VR/AR/MR, serious games, mobile learning, LMS environments, and blended or online approaches ( Radianti et al., 2020; Lee et al., 2024; Nantha et al., 2024). In immersive settings, engagement was frequently equated with increased attention, concentration, presence, and enjoyment ( Ibrahim et al., 2018; Liu et al., 2025). In game-based situations, engagement manifested itself as active involvement, interactivity, confidence and liking for the teaching format ( López-Fernández et al., 2023; Sharmin et al., 2024). Engagement, in particular, manifested as enhanced interaction, convenience and satisfaction ( Kaya & Adiguzel, 2021).

At the same time, the data indicate that engagement was often assessed qualitatively or via self-report rather than via validated behavioral indicators of long-term engagement. This limitation is particularly pronounced in the generative AI meta-analysis which reveals strong cognitive and affective effects, but surprisingly small behavior change ( Liu & Zhong, 2025; Li et al., 2025). A homologous problem arises in serious game and VR research, where short-term motivation and attention are common measures, but enduring behavioral change and long-term transfer are infrequently addressed ( Lee et al., 2024; Radianti et al., 2020). Thus, the review shows that technology consistently leads to higher engagement, yet there is a need in the field for more robust measures of sustained participation, transfer, and longer term behavioral outcomes.

### Challenges and facilitators in implementation

The synthesis revealed common structural and pedagogical challenges and critical facilitators that predicted positive course development outcomes (see Table 4).

Challenges at the level of the institution as whole, pedagogy, educators and students, and the technical, evaluative, ethical, and contextual dimensions are now becoming apparent as immersive and cutting-edge educational technology are being rapidly incorporated into higher education. Commonly reported institutional and infrastructure challenges are lack of sufficient internet bandwidth, power outages, high cost of hardware and HMD, disparity in the types of devices owned by students (PC or smartphones), and environmental limitations in conducting VR/AR sessions in real classrooms (temperature, noise), which hinder real-world classroom application (Alhazza & Yan, 205; Radianti et al., 2020; Loughlin et al., 2021; Sarrab et al., 2016; Alfauzan & Tarchouna, 2017; Biggs, 1996). The studies addresses these challenges with focused facilitators, such as ICT investments and strategies, power backup measures, procurement policies prioritizing accessible devices (mobile-first), technical support personnel, LMS integration, and monitored lab environments for immersive experiences (Alhazza & Yan, 205; Radianti et al., 2020).

Instructional-design and pedagogical issues include poor incorporation of learning theory (e.g., Constructive Alignment), disjointed course design, and too much focus on novelty or usability rather than on objectively assessable learning outcomes, such as ambiguous congruence between TLA (teaching-learning activities), ILO (intended learning outcomes), and assessment ( Balanyk, 2017; KOÇ, 2020; Ali, 2018; Mishra & Koehler, 2006). Good practitioners stress use of pedagogical models such as TPACK, Constructive Alignment, and ADDIE, scaffolded iterative course development, genuine problem-solving activities, clear ILO-TLA-assessment coherence, and formative review loops ( Mishra & Koehler, 2006; Pereira et al., 2024; Dilaines et al., 2024).

The preparations of the staff are often limited by the lack of technological instructional skills, low levels of TPACK, resistance to change, fear of making mistakes, and busy schedule that limit their time to develop designs ( Durdu & Dag, 2017; Njuguna, 2020; Mdodana-Zide, 2024; Bergroth-Koskinen & Seppälä, 2012). Facilitators include professional learning (e.g., sustained 12-week programs), reflective practice, micro-teaching, peer collaboration, mentoring, and mentoring models for faculty have been identified as facilitators for expanding instructional capacity and uptake ( Durdu & Dag, 2017; Mdodana-Zide, 2024).

Challenges related to student preparedness, including digital literacy disparities, variable background knowledge, cognitive overload from inadequately scaffolded multimodal content, novelty effects, and hardware differences impacting user experience, impede learning improvements ( Christodoulou & Angeli, 2022; Tamim et al., 2011; Ibrahim et al., 2018; Ruge et al., 2019). Facilitating strategies include orientation and onboarding processes, modular and sequenced content to reduce cognitive load, adaptive and personalized learning pathways, scaffolding with stepwise feedback, and mobile-friendly design ( Christodoulou & Angeli, 2022; Ruge et al., 2019).

Constraints such as delay, computational constraints, the limited haptic realism, usability constraints, the low reliability in prototypes, and the limited multimodal sensing fidelity of technology and system design limit the degree of fidelity and flexibility of educational environments ( Biggs, 1996; Treleaven & Voola, 2008; Wang et al., 2025; Han et al., 2025; Zhang et al., 2022). To better overcome these shortcomings, user-centered and participatory design, multimodal interaction, sensing enhancements, real-time feedback, iterative usability testing, and longitudinal follow-up are suggested ( Wang et al., 2025; Han et al., 2025).

Evaluations and generation of evidence are often short-term and varied, heavily relying on self-reporting focused on Level 1 to 2 outcomes rather than behavioral or longitudinal measures (Level 3 to 4) outcomes. Researchers have advocated for mixed-method, experimental, and longitudinal approaches, the linking of LA with performance data, larger sample sizes, and standardized metrics to enhance evidence ( Lee et al., 2024; Alfredo et al., 2024; Ford et al., 2017).

Issues of ethics, privacy, and trust, e.g., data privacy and consent, algorithmic bias, the opacity of GenAI and learning-analytics systems, and the potential for an over-reliance on outputs of AI, and the risk of over-reliance on outputs of AI, call for governance mechanisms. Enablers to be up the ladder recommended are human-centered design, ethical governance framework, explainability mechanism, stakeholders involvement with instructors oversight and explicit data policies ( Sajja et al., 2025; Alfauzan & Tarchouna, 2017; Maor, 2016).

Contextual and socio-economic factors, such as differences in device ownership and internet access, variations in technology usage at a regional level, along with limited adoption in the classroom, require mobile-accessible, low bandwidth options, adaptable delivery methods (blended/hybrid), contextualization to local learners, community collaboration, and supplementary financing ( Sarrab et al., 2016; Lutfi et al., 2022; Ali & Maliki, 2024). The literature as a whole showed that effective course development with emerging technologies requires substantive institutionally aligned support, pedagogically informed design, faculty development, learner-centered scaffolding, sound technical design, rigorous evaluation, ethical oversight, and sensitivity to context.

## Discussion

### Conceptual synthesis of relationship between key variables

**Figure 2.**
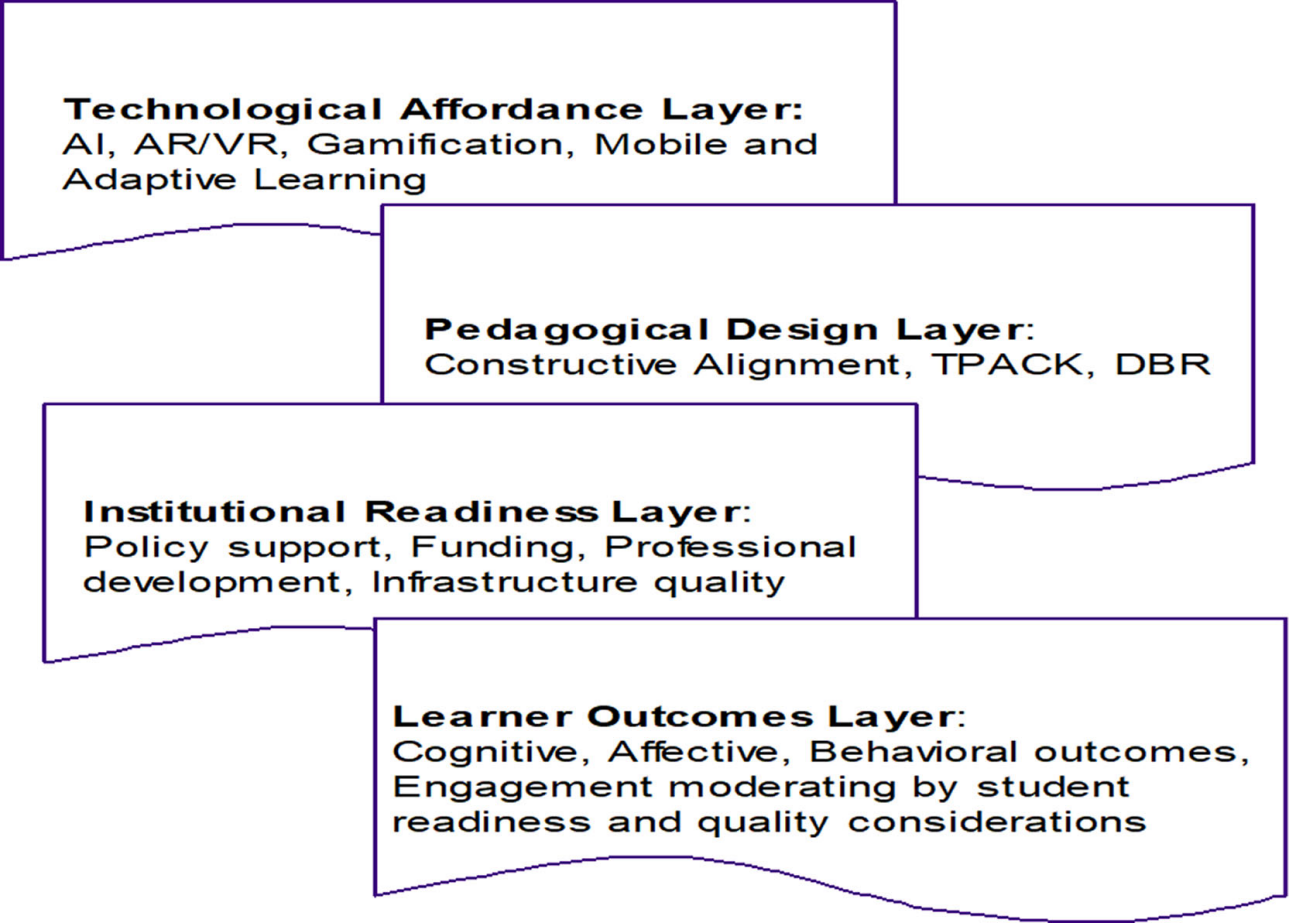
Technology-Integrated Learning and Course Development: Conceptual Model.

The notion of the socio-technical system (STS) is fundamental to how emerging technologies can and do find their way into higher education curricula. A socio-technical system is a unity of social (people, organizations, culture) and technical (mechanical, hardware, software) components ( Baxter & Sommerville, 2011). In the Process of ETICD (Emerging Technologies Integration for Course Development) in Higher Education, STS serves as an analytical lens for understanding how technological changes (e.g., AI, LA, virtual environments) are institutionalized within the educational ecosystems. In order to show the strong relationships between different facets related to success in enabling technologies for development of courses, the integrated outcome is represented at each level of multi-level model (see Figure 2) as follows:

### Interactions among design frameworks, technology types, and learning outcomes **as rooted in socio-technical system:** conceptual synthesis

The quantitative synthesis provides evidence for a dynamic interrelation between the level of the instructional design framework and the technology type, which affects the learning outcome in TEL environments. Positive effects on learning outcomes were shown for all major design frameworks with effect size estimates calculated from the review ranging from moderate to very large. TPACK, CA, ADDIE, and DBR all systematically yielded moderate-to-large gains in learning, indicating that the presence of a structured pedagogical design process continues to be a key determiner of instructional effectiveness regardless of the technological modality. The Deep Learning framework was in particular outstanding with the largest pooled effect size, showing that the most recent technologies, which include AI, combined with mixed-reality systems can greatly improve learning performance if these are arranged in meaningful instructional sequences. These results underlie the assumption that the effectiveness of technology is mediated through the pedagogical design of the instruction, and not by the level of technological complexity.

Figure 2 explains why thes findings are so sensible: technological affordances, pedagogical design processes, conditions of institutional readiness, and learner outcomes are all systemically interconnected. In this model, the Technological Affordances Layer is the context of the functional features embedded in the digital tool (e.g., immersion, personalization, automation, interactivity). The quantitative findings indicate that high-experience; high-adaptive feedback techno-stimulators (e.g., augmented reality, virtual reality, artificial intelligence, adaptive learning systems) are more effective than more conventional types of digital delivery platforms. For instance, immersive technologies exhibited the largest average effect size across technology types, which were attributed to the support for experiential learning, acquisition of procedural skills, and conceptual understanding. Such results are consistent with cognitive theory and models of experiential learning in which active participation and situated practice is a dynamic facilitator for improved knowledge retention and transfer.

The Pedagogical Design Layer of the model provides a lens into yet another process through which technological affordances are transformed into learning opportunities. It is suggestive of a clear cause in CA (and related models) of which the cause of optimal learning outcomes are “the explicit alignment between learning objectives, teaching and learning activities, and assessment methods”. When technologies were embedded in well-defined pedagogical sequences that involved scaffolding, formative feedback, and iterative refinement, studies are unanimous in reporting much more pronounced effects. In contrast, interventions that stressed technological novelty rather than systematic instructional design had relatively weaker or inconsistent effects on learning gains. This pattern bolsters the theoretical proposition that instructional design serves as the mediating through which technological potential is translated into educational effectiveness.

Regarding organizational and institutional readiness, before the commencement of investigation on digital instructional practice, infrastructure, and human resources, institutional readiness was found to be a critical enabling factor affecting the implementation of technology-enhanced instruction. The review revealed the persistence of institutional barriers such as limited infrastructure, inadequate technical support, and a lack of sufficient professional development, which limited technology integration and teaching effectiveness. In contrast, an institution led by a culture that provided resources including digital infrastructure for students and faculty, as well as faculty training and technical support manifested more robust and sustainable learning outcomes. This is in line with Institutional Readiness Layer in the conceptual model, inferring that the organizational capacity and leadership is instrumental to the systemic adoption of educational technologies. The relationship between institutional resources and pedagogical design processes is a further indication of the need for attention to integrated, holistic approaches to institutional implementation rather than discrete technological initiatives.

The Learner Outcomes Layer of the conceptual model describes the complexity of learning outcomes and includes dimensions of learning related to cognitive, affective, and behavioral aspects. The quantitative results showed that cognitive outcomes (e.g., knowledge gain and skills) were the most frequently evaluated and had the strongest impact among the studies. Positive changes in affective outcomes, including motivation, satisfaction and engagement, were also reported consistently across technology categories, with the most prominent effects evidenced in immersive and game-based learning environments. However, behavioral results and long-term transfer were less often evaluated and exhibited more inconsistent or weaker results. This discrepancy indicates that existing evaluation methods are more focused on short-term performance rather than on longer-term behavioral change, which is a major gap in the evidence base. The model predictions and loop nature explains this limitation and suggests that on-going assessment in iterative design phases is needed to capture short-term engagement as an indicator of long-term learning outcomes.

The conceptual model is better illustrated by the complex interactions portrayed when considering the Technology Type x Instructional Duration. Results from the review show that the majority of longer duration interventions (i.e., more than two weeks) appeared to have larger effects on learning than short-duration interventions. Long exposure time enables students to get used to new technological instruments, increases their procedural fluency, and involves them in continuous practice, which enhances knowledge storage and knowledge transfer. The result supports the model’s focus on temporal dynamics and iterative refinement processes in pedagogical design cycle. Similarly, learner participants’ prior knowledge and level of digital literacy also moderated learning results, which coincides with cognitive load theory and the expertise-reversal effect. These moderating variables indicate that the effectiveness of a technique changes based not only on its inherent quality, but how well that pedagogical complexity matches the maturity of the learners.

The synthesis also illustrates the presence of engagement as a mediating variable linking the technological affordances and learning outcomes. Regardless of technology type, indicators of engagement (including attention, participation, persistence and motivation) all significantly improved after technology adoption. Immersive and game-based contexts had even more pronounced engagement effects, a result of their interactive and experiential nature.

From a theoretical standpoint, the integrated results provide robust support for the STS model that underpinned the conceptual model. Socio-technical theory states that the performance of an organization results from the joint optimization of its technological, human and organizational subsystems. The result of this review is that success in technology-enhanced learning environments seems to be contingent upon a systemic coordination of these factors. Technology innovation by itself does not guarantee enhanced learning results, but the effectuality of innovation is exhibited when technology is incorporated within pedagogically sound instructional designs supported by institutional infrastructure and aligned with learner needs. The multi-level nature of the conceptual model can hence be seen as an adequate representation of the ‘nested’ processes that influence student achievements in digitally mediated learning environments.

Taken together, these results indicate that technology-enhanced learning is best viewed as a systemic type of process, and not simply as a singular teaching intervention. The most profound learning gains occurred when technological tools were incorporated within well-organized instructional sequences, in a climate of institutional readiness, and when subject to ongoing evaluation through feedback loops. This systems-level analysis reframes the target of educational reform from technology adoption to instructional design quality and organizational capacity. In sum, prospective research needs to emphasize longitudinal designs, standardized outcome measurements, and assessment of implementation fidelity to bolster the empirical base for technology-enhanced course development.

## Conclusion

The evidence from this systematic review indicates that new and emerging technologies have the potential to effectively support course development if the implementation is informed by a coherent pedagogy design, and supported by the institutional and contextual factors. Instructional design models such as TPACK, CA, ADDIE, and DBR, were among those reviewed, and cumulatively, across the studies, these models resulted in moderate to large positive effects on student learning (pooled effect size ranging from d = 0.65 to d = 0.74). To the best of the authors’ knowledge, the largest effect has been identified in deep learning-based framework combined with artificial intelligence and mixed reality technologies (d = 1.30), demonstrating the significant potential of advanced intelligent/immersive technology in improving learning when integrated with pedagogical application. On the other hand, hybrid or loosely coupled models appeared to bring more modest effects, adding weight to the conclusion that the use of technology by itself does not lead to better educational results. Instead, the alignment of learning goals, instructional activities, and assessment methods continues to be a key factor in determining the effectiveness of instruction.

This review also reinforces that quality teaching is moderated by different kinds of technology in terms of effect on outcomes for engagement and learning. Immersive technologies (augmented reality, virtual reality, and mixed reality) had the greatest overall effect on learning outcomes with large mean effect sizes and high levels of engagement indicators. Likewise, AI, generative AI, and ALS (adaptive learning systems) achieved statistically significant gains in cognitive performance, learner motivation, and SLR (self-regulated learning) behaviors. Game-based learning technology and mobile learning technology also demonstrated positive effects on students’ engagement and learning achievement, however, their effects were more or less varied by instructional alignment or design quality. Gains in engagement were often reported, especially in affective and cognitive domains, albeit the information on sustained behavioral change and longer-term learning transfer was still scarce. This is characteristic of technology-enhanced learning environments, which appear to consistently yield positive short-term learning and motivation outcomes, but often need longer-term investigation to assess enduring educational outcomes.

Addressing the third research question, the synthesis revealed a small number of contextual, institutional, and pedagogical factors that have been repeatedly cited as either facilitators or barriers to successful technology integration in course development. Institutional readiness, faculty professional development, infrastructure reliability, learner digital literacy, and ethical governance mechanisms were identified as key enablers. In contrast, prevalent barriers were poor connectivity, inadequate instructional design expertise, brief intervention lengths, and unequal access to digital materials. The conceptualization conceived in this review demonstrates how TEC development sits within a socio-technical system, where technological affordances, pedagogical design, institutional preparedness and learner attributes are all interacting and contributing to learning outcomes and achievement. Thus, effective practice involves more than technology, and having the other system components that align and function in holistic manner is necessary for effective adoption of technology in education.

## Implications

The results of this review will have significant consequences for practice, policy, and research related to TEC development. Educators and course designers, first and foremost, should emphasize pedagogical integration as opposed to technological innovation. The consistent moderate-to-large effect sizes observed for models such as TPACK, CA, or ADDIE indicate that structured instructional design is needed to transform technological affordances into observable learning gains. Hence, institutions should treat evidence-based design models as a best practice in course design and support full transparency among learning outcomes, teaching-learning activities, and assessment measures. Such alignment fosters not only instructional coherence, but also reliability and replicability of learning outcomes across different educational settings.

Second, institutions need to make strategic investments in its faculty professional development and teaching support services that assist in building their faculty’s digital teaching competencies. The review found that the most significant enabler for the integration of technology was the faculty’s readiness, and this was positively influenced by ongoing professional development programs, mentorship models, and collegial design practices. In particular, training interventions should be aimed in increasing educators’ ability to scaffold learning, offer adaptive feedback, and regulate cognitive load in technology enriched learning environments. More may be gained by building institutional capacity in these areas than by investing unquestioningly in additional hardware or software.

Third, the results demonstrate the necessity for system-level and long-term planning to facilitate the sustainable adoption of nascent technologies. Infrastructure dependability, fair access to devices, to connectivity, and to technical support services were cited as fundamental prerequisites for effective technology-enhanced learning. Investments in resilient digital infrastructure, in learning solutions that can be delivered in low bandwidth and through mobile devices, and in inclusive technology policies that address socioeconomic barriers to access should be the top priorities for policymakers and institutional leaders. Such investments tend to be critical in under-resourced educational settings where infrastructure constraints can severely attenuate the impact of technology-based interventions.

Fourth, researchers should broaden the evaluative lens to include behavioral, longitudinal, and organizational-level proxies of learning effectiveness rather than a focus on temporally-limited cognitive outcomes. The review identified chronic underreporting in the measurement of sustained behavior change, transfer of knowledge and long-term academic achievement. We encourage researchers to utilize mixed-method and longitudinal research designs, standardized measurement frameworks, and LA platforms and systems of systems to produce more robust and generalizable evidence concerning the long-term effects of TEL environments. In addition, future research should also investigate cost-effectiveness, potential for scale, and context-specific adaptability to inform evidence-based decision-making in educational policy and practice.

In the end, ethical considerations around technology use and application should be woven into the technology-enhanced course at all levels. As AI and LA systems become increasingly common in education, the associated issues around data privacy, algorithmic bias, transparency, and learner autonomy are likely to escalate. Institutions ought to define clear data governance policies, ensure transparency in algorithmic decision-making, and uphold human agency in technology-mediated instruction. The ethical and equitable use of educational technologies is critical to build trust with stakeholders and to maintain the long-term innovation of learning systems.

## Data Availability

All data supporting the findings of this study are openly available in the Zenodo repository under an open-access license (CC0): https://doi.org/10.5281/zenodo.19732174 Citation: Geleto, L., Abame, A., Lolamo, A., Bekene, T., & Bekele, J. (2026). Full Data Sets and References for Systematic Review [Data set]. Zenodo. https://doi.org/10.5281/zenodo.19732174 The following materials have been deposited:
✓ Completed PRISMA checklist ✓ PRISMA flow diagram ✓ Study screening and selection dataset ✓ Extracted data used for analysis ✓ Variables and coding framework ✓ Values underlying figures and tables
