# Supplementary material for: Emerging Technologies in Higher Education Course Development: A Systematic Review of Design Frameworks, Learning Outcomes, and Pedagogical Integration
Source: F1000Res. 2026 Jun 1;15:716. Originally published 2026 May 12. [Version 2] doi: 10.12688/f1000research.179684.2 (PMC13231138; doi:10.12688/f1000research.179684.2)
Supplement: Peer review history

# Reviewer report: Reviewer response for version 2

**DOI:** 10.5256/f1000research.202024.r485050

**Published:** 2026-06-02

**Authors:** Louis Robert C. Sison

**Affiliations:** 1 Bulacan State University, Bulacan, Philippines

1. The introduction clearly establishes the need to examine emerging technologies in higher education course development, and the research questions are explicitly stated. The paper also situates the review within pedagogical frameworks such as TPACK, ADDIE, Constructive Alignment, and DBR. 

 2. The paper provides databases, search strings, eligibility criteria, PRISMA screening, data extraction procedures, and MMAT appraisal. However, replication would be stronger if the authors further clarified reviewer screening procedures, discrepancy resolution, and the validation process for NotebookLM-assisted extraction.

 3. The descriptive quantitative synthesis is useful, but is should not be interpreted as a formal meta-analysis. The paper averages effect sizes without weighting by sample size or variance and does not report heterogeneity, confidence intervals, or publication bias. Therefore, the findings should be framed as indicative trends rather than definitive effect estimates.

 4. The conclusions are generally supported because the results consistently show that emerging technologies are most beneficial when aligned with sound pedagogy, institutional readiness, and learner needs. However, conclusions about "strongest effects" should be softened because the quantitative results are descriptive rather than meta-analytic. 

 Generally, the paper is scientifically acceptable and contributes meaningfully to technology-enhanced higher education research, but it still has important reservations, especially regarding the framing and interpretation of the quantitative synthesis.

Are the rationale for, and objectives of, the Systematic Review clearly stated?

Yes

Is the statistical analysis and its interpretation appropriate?

Partly

If this is a Living Systematic Review, is the ‘living’ method appropriate and is the search schedule clearly defined and justified? (‘Living Systematic Review’ or a variation of this term should be included in the title.)

Not applicable

Are sufficient details of the methods and analysis provided to allow replication by others?

Partly

Are the conclusions drawn adequately supported by the results presented in the review?

Yes

Reviewer Expertise:

Teacher Education, Educational Technology, Mathematics Education

I confirm that I have read this submission and believe that I have an appropriate level of expertise to confirm that it is of an acceptable scientific standard, however I have significant reservations, as outlined above.

# Reviewer report: Reviewer response for version 2

**DOI:** 10.5256/f1000research.202024.r485046

**Published:** 2026-06-02

**Authors:** Alexandhrea Hiedie Dumagay

**Affiliations:** 1 Western Mindanao State University, Zamboanga City, Philippines

This manuscript addresses a timely and relevant issue in higher education by reviewing the integration of emerging technologies in course development and pedagogical practice. The topic is important given the growing use of artificial intelligence, immersive technologies, learning analytics, and digital instructional tools across higher education institutions. The manuscript also provides a broad overview of instructional design frameworks, including TPACK, Constructive Alignment, ADDIE, and Design-Based Research, and attempts to relate these frameworks to learning outcomes and pedagogical effectiveness.

 One of the strengths of the paper lies in its wide literature coverage and its effort to synthesize findings from multiple technology domains within a single review. The discussion of design frameworks and their relationship to learning outcomes is particularly useful, as is the attempt to interpret the findings through a socio-technical systems perspective. The inclusion of institutional, pedagogical, and learner-related factors further strengthens the discussion by acknowledging that technology integration depends on more than technological availability alone.

 At the same time, some aspects of the review would benefit from further clarification. Although the manuscript states that PRISMA procedures were followed, the methodological reporting could be strengthened. In particular, the procedures used for assessing study quality and risk of bias are not clearly described. Since the review includes 76 studies with diverse methodologies and contexts, additional explanation regarding how study quality and heterogeneity were addressed would improve confidence in the synthesis.

 The organization of the manuscript could also be improved in some sections. Discussions of technology benefits, engagement outcomes, and implementation challenges occasionally overlap and repeat similar ideas. Greater condensation and clearer transitions may help improve readability and maintain the flow of argument. In addition, the early discussion of the Ethiopian policy and higher education context is informative, although its connection to the later synthesis and conclusions could be made more explicit.

 The paper appropriately highlights the importance of pedagogical alignment, institutional readiness, and faculty competence in successful technology integration. However, the manuscript itself notes the limited availability of longitudinal and behavioral evidence, and this limitation may deserve stronger emphasis in the discussion and conclusion. While many studies report positive engagement and cognitive outcomes, longer-term impacts and transfer effects appear less consistently documented.

 Overall, this manuscript addresses an important topic and offers a potentially valuable synthesis of research on emerging technologies and higher education course development. The paper could make a useful contribution to discussions of technology-enhanced learning and instructional design.

Are the rationale for, and objectives of, the Systematic Review clearly stated?

Yes

Is the statistical analysis and its interpretation appropriate?

Yes

If this is a Living Systematic Review, is the ‘living’ method appropriate and is the search schedule clearly defined and justified? (‘Living Systematic Review’ or a variation of this term should be included in the title.)

Not applicable

Are sufficient details of the methods and analysis provided to allow replication by others?

Yes

Are the conclusions drawn adequately supported by the results presented in the review?

Yes

Reviewer Expertise:

Education, AI in Education, Educational Technology

I confirm that I have read this submission and believe that I have an appropriate level of expertise to confirm that it is of an acceptable scientific standard.

# Reviewer report: Reviewer response for version 1

**DOI:** 10.5256/f1000research.198223.r485048

**Published:** 2026-05-18

**Authors:** Serafeim A. Triantafyllou

**Affiliations:** 1 Aristotle University of Thessaloniki, Thessaloniki, Greece

The manuscript addresses an important and timely topic concerning the integration of emerging technologies into higher education course development. The review is broad in scope and provides useful synthesis regarding instructional design frameworks, learning outcomes, and institutional factors influencing implementation. The inclusion of frameworks such as TPACK, Constructive Alignment, ADDIE, and DBR adds conceptual coherence to the discussion.

 The introduction is comprehensive and clearly motivates the review. The research questions are explicit and relevant. The PRISMA-based structure strengthens transparency, and the manuscript includes substantial descriptive synthesis of technology types, pedagogical models, and implementation challenges.

 However, there are methodological concerns that limit the rigor of the quantitative synthesis. Although the manuscript reports “pooled” effect sizes, the statistical aggregation procedures do not constitute a formal meta-analysis. Effect sizes appear to be averaged without weighting by sample size or variance, and there is no assessment of heterogeneity, confidence intervals, publication bias, or study quality/risk of bias. These omissions weaken the strength of the statistical conclusions. The authors should either frame the quantitative synthesis more cautiously as descriptive aggregation or conduct a more rigorous meta-analytic procedure.

 The methods section would also benefit from greater detail to improve reproducibility. Specifically, the manuscript should provide complete database search strings, screening procedures between reviewers, inter-rater agreement measures, and explicit criteria for study quality appraisal. The use of NotebookLM during extraction should also be clarified further, including how accuracy and reliability were ensured.

 Some sections contain language and formatting inconsistencies, typographical issues, and citation irregularities that require careful proofreading. Tables are informative, but some effect-size calculations and terminology should be clarified for consistency.

 Despite these concerns, the manuscript contributes meaningfully to the literature on technology-enhanced learning and provides useful conceptual synthesis for researchers and practitioners.

Are the rationale for, and objectives of, the Systematic Review clearly stated?

Yes

Is the statistical analysis and its interpretation appropriate?

Partly

If this is a Living Systematic Review, is the ‘living’ method appropriate and is the search schedule clearly defined and justified? (‘Living Systematic Review’ or a variation of this term should be included in the title.)

Not applicable

Are sufficient details of the methods and analysis provided to allow replication by others?

Yes

Are the conclusions drawn adequately supported by the results presented in the review?

Yes

Reviewer Expertise:

Educational Technology, Gamification, Computational Thinking, Intelligent Tutoring Systems, Artificial Intelligence in Education, Didactics

I confirm that I have read this submission and believe that I have an appropriate level of expertise to confirm that it is of an acceptable scientific standard, however I have significant reservations, as outlined above.
